# Polymorphism of A133S and promoter hypermethylation in Ras association domain family 1A gene (*RASSF1A*) is associated with risk of esophageal and gastric cardia cancers in Chinese population from high incidence area in northern China

**DOI:** 10.1186/1471-2407-13-259

**Published:** 2013-05-25

**Authors:** Sheng Li Zhou, Juan Cui, Zong Min Fan, Xue Min Li, Ji Lin Li, Bao Chi Liu, Dong Yun Zhang, Hong Yan Liu, Xue Ke Zhao, Xin Song, Ran Wang, Ze Chen Yan, Hui Xing Yi, Li Dong Wang

**Affiliations:** 1 Henan Key Laboratory for Esophageal Cancer Research, The First Affiliated Hospital of Zhengzhou University, Zhengzhou, Henan 450052, China; 2 Cancer Research Center, Xinxiang Medical University, Xinxiang, Henan 453003, China; 3 Department of Pathology, Cixian Hospital, Cixian, Hebei 056500, China; 4 Department of Pathology, Linzhou Esophageal Cancer Hospital, Linzhou, Henan 456500, China; 5 Department of Surgery, Shanghai Public Health Clinical Center Affiliated to Fudan University, Shanghai 201508, China; 6 Henan Medical Genetics Institute, Henan People’s Hospital, Zhengzhou University, Zhengzhou, Henan 450003, China; 7 Department of Urology, The First Affiliated Hospital, Zhengzhou University, Zhengzhou, Henan 450052, China

**Keywords:** Esophageal squamous cell carcinoma, Gastric cardia adenocarcinoma, A133S in *RASSF1A*, Polymorphism, Methylation, Protein expression

## Abstract

**Background:**

The role of tumor suppressor gene *RASSF1A* in the esophageal and gastric cardia carcinogenesis is still inconclusive. In this study, the polymorphism, promoter methylation and gene expression of *RASSF1A* were characterized in esophageal squamous cell carcinoma (ESCC) and gastric cardia adenocarcinoma (GCA).

**Methods:**

We firstly analyzed the prevalence of *RASSF1A* A133S in a total of 228 cancer patients with ESCC (n=112) and GCA (n=116) and 235 normal controls by polymerase chain reaction (PCR) and restriction enzyme-digestion assay. Then, the promoter methylation status of the *RASSF1A* in ESCC (n=143), GCA (n=92) and corresponding adjacent normal tissues were further investigated using methylation-specific PCR (MSP) approach. Finally, the RASSF1A protein expression were determined in ESCC (n=27), GCA (n=24) and the matched adjacent normal tissues by immunohistochemical method.

**Results:**

The frequency of 133Ala/Se and Ser/Ser genotype was significantly higher in GCA patients than in normal controls (19.0% vs. 10.2%, *P*=0.02). Compared with Ala/Ala genotype, Ala/Se and Ser/Ser genotype significantly increased susceptibility to GCA (OR=2.06, 95% CI=1.09–3.97). However, this polymorphism had no association with ESCC (*P*=0.69). The promoter methylation of *RASSF1A* gene was significantly increased the risk to both ESCC (OR=5.90, 95% CI=2.78–12.52) and GCA (OR=7.50, 95% CI= 2.78–20.23). Promoter methylation of *RASSF1A* gene in ESCC was also associated with age and cancer cell differentiation (for age: OR=3.11, 95% CI=1.10–8.73; for differentiation: OR=0.29, 95% CI=0.12–0.69). RASSF1A positive expression was significantly decreased the risk of GCA (OR=0.16, 95% CI=0.03–0.83). In contrast, there was no statistical significance between RASSF1A positive expression and ESCC. The expression of RASSF1A protein trend to be positively related with older GCA patients (OR=16.20, 95% CI=1.57–167.74).

**Conclusions:**

The present findings suggest that alterations of *RASSF1A* may play an important role in gastric cardia carcinogenesis in terms of polymorphism, promoter hypermethylation and protein expression. Whereas, *RASSF1A* hypermethylation may probably also be involved in esophageal squamous cell carcinogenesis.

## Background

Esophageal squamous cell carcinoma (ESCC) remains the main predominant histological type of esophageal cancer and the leading cause of cancer-related deaths in China [1,2]. ESCC has a striking geographic distribution in China, with higher prevalence in some areas of China, especially in Taihang Mountain areas of Henan, Hebei and Shanxi provinces [3], where nutritional deficiencies, intake of pickled vegetables, nitrosamine-rich or mycotoxin-contaminated foods and low socioeconomic status are likely to contribute to ESCC [4]. Also, in these high-risk areas there is a strong tendency toward familial aggregation of ESCC [5], suggesting that genetic susceptibility, in combination with exposure to environmental risk factors, contributes to the high rates of ESCC in these areas [6]. ESCC has been generally recognized as a multi-stage progression process, in which multiple genetic and epigenetic alterations may be involved. Recent genome-wide association study (GWAS) for ESCC has indicated that the tumor suppressor gene of Ras association domain family 1A gene (*RASSF1A*) may be associated with high risk to ESCC [6]. *RASSF1A* locates at 3p21.3 and participates in regulating cell cycle, apoptosis, microtubule stability and other physiological activities [7,8]. Accumulated evidences have indicated the possible crucial role of *RASSF1A* methylation on esophageal carcinogenesis in Chinese population, the methylation rates varied from 14.9% [9] in Beijing with a low incidence for ESCC, to 48.5% [10] in Hangzhou with a higher incidence for ESCC, indicating the disparity of *RASSF1A* methylation with different environment background. The single nucleotide polymorphism (SNP) Ala133Ser (A133S) in *RASSF1A* has been reported to be involved in the lung and breast cancer [11,12]. Linzhou city (formerly Linxian) in Henan province has been well documented as the highest incidence area for ESCC in China [2,3]. However, the effects of *RASSF1A* polymorphism and methylation on esophageal carcinogenesis have not been well characterized in the population from this highest incidence area in China.

Gastric cardia adenocarcinoma (GCA), with its epicenter located between 1 cm proximal and 2 cm distal of the esophago-gastric junction [13], is another common cancer in China, which bears many similarities to ESCC in terms of concurrent geographic distribution and environmental risk factors [6,14]. It is reasonable to clarify the molecular profile of ESCC and GCA, which would be helpful to identify molecular biomarkers for high risk subject screening and early detection for these two diseases. Thus, the present study was undertaken to determine the effect of *RASSF1A* polymorphism, promoter methylation status and protein expression on esophageal and gastric cardia carcinogenesis in patients from the high incidence area for both ESCC and GCA in Linzhou city, Henan province, northern China.

## Methods

### Study population

A total of 259 patients were recruited in this study, including 143 ESCC (82 males with a mean age (average±standard deviation) of 57±10 years and 61 females with a mean age of 59±10 years) and 116 GCA (70 males with a mean age of 55±10 years and 46 females with a mean age of 60±10 years). All the patients were from Linzhou, the high incidence area for both ESCC and GCA. All the patients were performed surgical treatment, without chemotherapy and/or radiotherapy before the surgery. In addition, 235 normal control subjects were enrolled in this study, including 115 males with a mean age of 58±9 years and 100 females with a mean age of 59±9 years. All the subjects were performed biopsy under endoscopy to exclude upper gastrointestinal tumor and questionnaires to exclude tumor history and tumor family history. All these subjects were from endoscopy screening for early cancer detection on symptom-free subjects in Linzhou city. Informed consents were obtained from all participants according to Zhengzhou University and Linzhou Esophageal Cancer Hospital Review Boards.

### Histopathological examinations

Histopathlogical examinations and TNM staging were performed by two pathologists (X. M. Li and J. L. Li) based on the UICC criteria in 2002 [15]. In brief, all the esophageal cancers were confirmed as squamous cell carcinoma and gastric cardia cancers as adenocarcinoma. The gross morphological types for ESCC and GCA were classified as medullary, fungating, ulcerating, constriction and intraluminal in ESCC; and protruding, ulcerating and infiltrating in GCA. The differentiation for both ESCC and GCA was classified as high, middle and low grades. Lymph node metastasis was recorded as negative and positive, if any.

### Blood sample and surgically resected ESCC and GCA tissue collection

Five ml peripheral blood samples for each patient and each normal subject, and surgically resected ESCC and GCA tissue samples were collected sequentially at Linzhou Esophageal Cancer Hospital and endoscopy screening from 2000 to 2008. The blood samples were stored at −40°C until use. The tumor tissues and matched normal tissues were collected after surgery, half of the surgically resected sample was formalin-fixed, paraffin-embedded and another half stored at −80°C. The criteria for matched samples are defined as that the tumor tissues and the normal tissues are from the same patients, and the matched normal tissues are taken at the surgically resected margin excluding the infiltration of carcinoma cells. Finally, a total of 463 blood samples were recruited for polymorphism detection, including 112 ESCC, 116 GCA and 235 normal controls. Another group of surgically resected esophageal and gastric cardia cancer and matched normal tissue specimens were recruited for the *RASSF1A* methylation detection. A total of 143 ESCC tissue specimens and 92 GCA tissue specimens were used for methylation detection, including 62 matched esophageal cancer and normal tissues, and 30 matched gastric cardia cancer and normal tissues. For further determination of RASSF1A protein expression, 27 ESCC tissue specimens with 27 matched normal esophageal tissue specimens and 24 GCA tissue specimens with 24 matched normal gastric cardia tissue specimens were recruited from 143 ESCC and 92 GCA, which had been used for *RASSF1A* methylation detection. This study was reviewed and approved by the Institute Research Ethics Committee of the Zhengzhou University and informed consents were obtained from all participants before their blood and tissue samples were used.

### DNA extraction

For polymorphism detection, genomic DNA from peripheral leucocytes was extracted by phenol/chloroform extraction method, dissolved in TE balanced solution, and then stored at −80°C for next procedure.

For methylation profiles and protein expression examination, the paraffin-embedded tissue blocks of tumor tissue (both ESCC and GCA) and corresponding adjacent normal tissue were sectioned for hematoxylin and eosin staining with the purpose of identifying pathological diagnose reviewed by pathologist. Regions with neoplastic compositions of 80% or greater were marked as tumor tissue under optical microscope. Depending on the size of the tissue, 15 to 20 consecutive 10 μm sections of each block were manually microdissected using a needle and collected into 1.5 ml microtubes for DNA extraction. The tissue DNA extraction was carried out using the Puregen Genomic DNA purification kit (Gentra Systems, MN, USA) according to the manufacturer’s protocols. The DNA from tissue was stored at −80°C for next procedure.

### Genotyping

The nest PCR was performed in a 25 μl reaction containing 1 μl for genome DNA, 10mmol/L dNTP, 10X buffer solution, 5pmol up and down primer respectively and 1U Ex Taq DNA polymerase. All the reagents involved were purchased from TaKaRa, Beijing city, China. The sequences of both outside and inside primers were designed based on the reference sequence (chr3-50342222-50353371) using Primer 3.0 as follows: outside primer: forward 5′ATG ATT CTG TCT TTC CCT TAT CCA and reverse 5′ACC AAA CCT TGA TAA TAG GTT CCA; inner primer: forward 5′AAG GCA GTC AGT TTC CAA AGA CT and reverse 5′ATG AAG AGG TTG CTG TTG ATC TG. The amplification was conducted on the GennAmp RCR system 9700 gene amplifier (ABI, California, USA) under the following thermo-cycler conditions: pre-degeneration at 94 °C for 5 min; followed by 16 cycles at 94°C for 30 sec, at 64°C (−0.3°C/circulation) 30 sec and at 72°C for 30 sec; 20X (94°C for 30 sec, 56°C for 40 sec and 72°C for 30 sec); then, extension at 72°C for 10 min, and finally stored at 4°C. The final product of nest PCR was 194 bp. 10 μl PCR product was digested with restriction enzymes AluI (TaKaRa, Beijing city, China.) incubated at 37°C overnight. The digestion product was detected on a 3% agarose gel stained with ethidium bromide under UV illumination.

**Figure 1:**
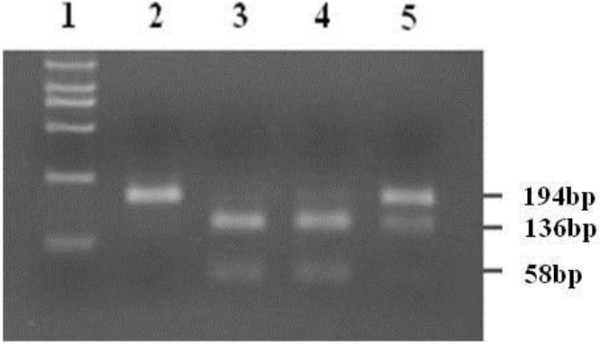
**The genotying results of the *RASSF1A *SNP at codon 133.** PCR analysis of the *RASSF1A* SNP at codon 133. Land 1: Marker; Land 2: Ser/Sergenotype; Land 3 and 4: Ala/Ala genotypes; Land 5:Ala/Ser genotype.

The 133rd amino acid which was encoded by the exon 3 of *RASSF1A* is GCT (Ala) or TCT (Ser), if the locus was allele G, this read was restriction site of AluI. Because of that, Ala/Ala genotype was two bands of 136 bp and 58 bp, Ala/Ser genotype was three bands of 194 bp, 136 bp and 58 bp, Ser/Ser genotype was just one band of 194 bp (Figure 1). For quality assurance, both Ala/Ser and Ser/Ser genotypes were genotyped more than twice, moreover, all of the Ser/Ser, portion of Ala/Ser and some Ala/Ala genotypes which randomly selected were sequenced to testify the genotyping results. The sequencing analysis was conducted using ABI3730XL Sequencers (ABI, California, USA) at Beijing Sunbiotech Co., Ltd. (Beijing, China).

### Bisulfite treatment and methylation specific PCR (MSP)

Genomic DNA (1μg) was treated with sodium bisulfite modification in order to converting unmethylated cytosines to uracils using the CpGenome™ DNA Modification Kit (S7820) (Chemicon, California, USA) according to manufacturer’s protocol. The modified DNA was purified by using a Wizard DNA Clean-Up System (Promega Corporation, Madison, USA) following manufacturer’s protocol.

**Figure 2:**
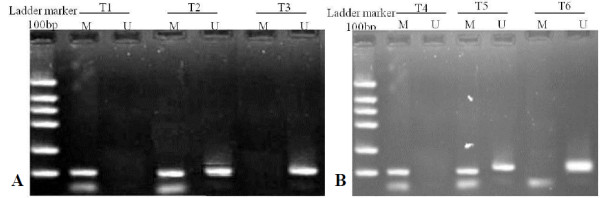
**MSP analysis of *RASSF1A *gene in ESCC and GCA tissue.** Representative MSP results of three ESCC tissues (**A**, T1, T2, and T3) and (**B**) was the results of three GCA tissues (T4, T5, and T6). Lane M: indicates the presence of methylated genes; Lane U: indicates the presence of unmethylated genes. T1 and T4 were fully hypermethylation which revealed 93 bp band (M) with hypermethylated primers; T3 and T6 were unmethylation, having only unmethylated band of 105 bp; T2 and T5 were Hemi-methylation with both hypermethylated band and unmethylated band.

The bisulfite-modified DNA was subjected to methylation specific polymerase chain reaction (MSP) as described previously [16]. Primers targeting promoter region of RASSF1A were as follows: The specific primers for methylated sequences (forward 5′- GTG TTA ACG CGT TGC GTA TC and reverse 5′- AAC CCC GCG AAC TAA AAA CGA) and for unmethylated sequences (forward 5′- TTT GGT TGG AGT GTG TTA ATG TG and reverse 5′- CAA ACC CCA CAA ACT AAA AAC AA), which generates PCR products of 93 and 105 bp, respectively. The total 25 ml of PCR mix contained 50-100ng bisulfite-modified DNA, 10X PCR buffer (Mg^2+^ Plus), 3.0 μl; 2.5 mM dNTPs, 3.0 μl; 10 μM of each primer, 2.0 μl; and 0.5 U *Taq*^HS^ DNA polymerase, 2.5 μl (TaKaRa, Dalian city, China). The amplification was conducted on the Tgradient RCR system (Biometra, Goettingen, German) under the following thermo-cycler conditions: pre-degeneration at 95°C for 15 min; followed by 40 cycles at 95°C for 55 sec, at 65°C for 55 sec and at 72°C for 1 min; 20X (94°C for 30 sec, 56°C for 40 sec and 72°C for 30 sec; then, extension at 72°C for 10 min. The placenta tissue DNA which was treated with Sss I methyltransferase (TaKaRa, Dalian city, China) was used as positive control for methylation, while taking the placenta tissue DNA which was not digested by Sss I methyltransferase as positive control for unmethylation; Distilled water was used as negative control for PCR. Both of the positive and negative controls were deal with in the same procedures. Six μl of PCR products were separated on 10% polyacrylamide gel. The gel was then stained with ethidium bromide, and visualized under UV illumination. Methylated samples were defined as the presence of methylated PCR products in those samples (Figure 2).

### Immunohistochemical analysis

Partial of the samples which were inspected by MSP, about 27 ESCC and 24 GCA, as well as corresponding adjacent normal epithelial tissues were subjected to investigate RASSF1A protein expression by immunohislochemical staining. Immunohislochemical analysis was performed using the avidin-biotin-peroxidase complex method as previously described [17]. The rat anti-human monoclonal RASSF1A antibody (eBioscience corporation, San Diego, USA) was used at 1:500 dilution. Intense nuclear or cytoplasm staining was the criterion for a “positive” reaction. We applied the criteria established by our laboratory previously [17] to describe the types of positive result as follows: “scattered”, in which only some isolated positive cells were identified; “papillary”, where immunostain-positive cells were identified only in the papillary area; “focal”, where wide clusters of positive cells were seen in some areas of the epithelia; and “diffuse”, in which the sheets of positive cells were found throughout most areas of the lesions. Immunohistochemical labeling was estimated in an outcome-blinded model by two pathologists on a compound microscope.

### Statistical analysis

Consistency with Hardy–Weinberg equilibrium (HWE) of the genotypes of the ESCC/GCA and control groups was established by Chi-squared tests. The association of Genotype and diseases were assessed using logistic regression and expressed as odds ratios with 95% confidence intervals. For *RASSF1A* gene methylation and protein expression, the correlations with clinic characteristics in ESCC and GCA tissues were evaluated using multiple univariate logistic-regressions. The statistical analyzes were carried out with SPSS 17.0 software package. All tests were two tailed*. P* < 0.05 was considered statistically significant.

## Results

### Frequency of *RASSF1A* A133S in ESCC and GCA patients

All the 235 control, 112 ESCC and 116 GCA samples were analyzed for the presence of A133S (Table 1). There were no departures from the HWE in the genotyping results of ESCC, GCA or control samples (*P*=0.83). The homozygous or heterozygous for A133S were accounted for 11.6% (13/112) in the ESCC and 10.2% (24/235) in control subjects. Though the frequencies of A133S in ESCC was a little higher than in controls, Ala/Se and Ser/Ser genotype did not increase the risk of ESCC compared with Ala/Ala genotype (11.2% vs. 10.2%, *P*=0.69; OR=1.15; 95% CI=0.51–2.44).

**Table 1:** **The genotypings of A133S in *RASSF1A *gene on ESCC and GCA patients and normal controls**

Genotypings*	**Control**		**ESCC**					**GCA**				
	**n**	**(%)**	**n**	**(%)**	***P***	***OR***	***(95%CI)***	**n**	**(%)**	***P***	***OR***	***(95%CI)***
**Ala/Ala**	211	(89.8)	99	(88.4)				94	(81.0)			
**Ala/Ser**	23	(9.7)	11	(9.8)	0.96	1.02	(0.48–2.17)	18	(15.5)	0.09	1.76	(0.91–3.41)
**Ser/Ser**	1	(0.4)	2	(1.8)	0.24	4.26	(0.38–47.02)	4	(3.5)	**0.04**	9.01	(0.99–83.31)
**Ala/Se and Ser/Ser**	24	(10.2)	13	(11.6)	0.69	1.15	(0.51–2.44)	22	(19.0)	**0.02**	2.06	(1.09–3.97)

*: Ala/Ala: homozygous for wild-type codon 133, Ala/Ser: heterozygote for codon 133, Ser/Ser: homozygous for codon A133S.

Intriguingly, in GCA, there was a significant difference of the frequency of the *RASSF1A* A133S T allele compared with the controls (19.0% vs. 10.2%). The individuals carrying A133S (Ser/Ser) genotype had a much higher susceptibility to GCA compared with people carrying Ala/Ala genotype (*P*=0.04, OR=9.01, 95% CI= 0.99–83.31). In addition, compared with Ala/Ala genotype, Ala/Se and Ser/Ser genotype also significantly increased susceptibility to GCA (*P*=0.02, OR=2.06, 95% CI=1.09–3.971).

### The promoter methylation of *RASSF1A* in ESCC and GCA patients

The promoter methylation of *RASSF1A* in ESCC tissue was 3.4-fold higher than in adjacent normal mucosa (76/143, 53% vs. 10/62, 16%, *P *< 0.001). The promoter hypermethylation of *RASSF1A* gene significantly increased almost 6-fold higher the risk to ESCC development (OR=5.90, 95% CI=2.78–12.52). Interestingly, the similar results were observed in GCA, the promoter hypermethylation of *RASSF1A* gene significantly increased almost 7.5-fold higher the risk to GCA development (65% in GCA vs. 20% in adjacent normal mucosa, OR=7.50, 95% CI=2.78–20.23) (Table 2 and Figure 2).

**Table 2:** **The profiles of *RASSF1A *promoter hypermethylation in ESCC and GCA tissues and in normal tissues adjacent to the corresponding ESCC and GCA**

**Tissues**^**#**^	**N**	**Methylated**		**Unmethylated**		***P***	***OR***	***(95%CI)***
		**n**	**(%)**	**n**	**(%)**			
**ESCC**	143	76	(53)	67	(47)	**<0.001**	5.90	(2.78–12.52)
**ENOR**	62	10	(16)	52	(84)			
**GCA**	92	60	(65)	32	(35)	**<0.001**	7.50	(2.78–20.23)
**GNOR**	30	6	(20)	24	(80)			

^#^: ESCC: esophageal squamous cell carcinoma, ENOR: esophageal normal epithelial tissue, GCA: gastric cardia adenocarcinoma, GNOR: gastric cardia normal epithelial tissue.

Furthermore, the promoter methylation of *RASSF1A* in ESCC patients with the age group from 50–60 years old had a significantly higher risk to ESCC than in those with the age less than 50 years old (OR=3.11, 95% CI=1.10–8.73) (Table 3). The ESCC with moderate differentiation had a lower frequency of *RASSF1A* methylation than in those with higher differentiation (OR=0.28, 95% CI=0.12–0.69) (Table 3). However, multivariate analysis did not show did not show any significant association for *RASSF1A* promoter methylation and gender, gross pathologic classification, infiltration degree, lymph node metastasis and clinical stages both in ESCC and GCA.

**Table 3:** **Distribution of *RASSF1A *promoter methylation by clinicopathological classifications and protein expression in ESCC and GCA tissues**

**Classification**	**ESCC (n=143)**									**GCA (n=92)**								
	**N**		**Methylated**		**Unmethylated**		***P***	**OR**	**(95%CI)**	**N**		**Methylated**		**Unmethylated**		***P***	***OR***	***(95%CI)***
	**n**	**(%)**	**n**	**(%)**	**n**	**(%)**				**n**	**(%)**	**n**	**(%)**	**n**	**(%)**			
**Gender**																		
**Male**	82	(57)	43	(52)	39	(48)				58	(63)	37	(64)	21	(36)			
**Female**	61	(43)	33	(54)	28	(46)	0.84	0.94	(0.48–1.82)	34	(37	23	(68)	11	(32)	0.71	0.84	(0.34–2.07)
**Age (y)**																		
**≤50**	25	(17)	13	(52)	12	(48)				21	(23)	16	(76)	5	(24)			
**50–60**	48	(34)	37	(77)	11	(23)	**0.03**	3.11	(1.10–8.73)	32	(35)	19	(59)	13	(41)	0.21	0.46	(0.13–1.56)
**>60**	70	(49)	26	(37)	44	(63)	0.20	0.55	(0.22–1.37)	39	(42)	25	(64)	14	(36)	0.34	0.56	(0.17–1.85)
**Gross pathologic classification**^**Φ**^																		
**1**	38	(27)	18	(47)	20	(53)				17	(18)	10	(59)	7	(41)			
**2**	25	(17)	16	(64)	9	(36)	0.58	0.74	(0.25–2.20)	32	(34)	24	(67)	8	(33)	0.25	2.10	(0.60–7.36)
**3**	29	(20)	19	(66)	10	(34)	0.91	0.92	(0.23–3.77)	33	(36)	21	(64)	12	(36)	0.74	1.23	(0.37–4.06)
**4**	19	(13)	15	(79)	4	(31)	0.06	0.39	(0.14–1.05)	ND	ND	ND	ND	ND	ND			
**Unclear**	32	(22)	8	(25)	24	(75)				10	(11)	5	(50)	5	(50)			
**Differentiation classification**																		
**High**	32	(22)	22	(69)	10	(31)				20	(22)	14	(70)	6	(30)			
**Middle**	70	(49)	27	(39)	43	(61)	**0.006**	0.28	(0.12–0.69)	31	(34)	22	(71)	9	(29)	0.94	1.05	(0.31–3.59)
**Low**	34	(24)	26	(76)	8	(24)	0.48	1.48	(0.50–4.39)	41	(45)	24	(59)	17	(41)	0.39	0.61	(0.19–1.89)
**Unclear**	7	(5)	1	(14)	6	(86)				ND	ND	ND	ND	ND	ND			
**Tumor stage (T) classification**																		
**T1+T2**	25	(17)	13	(52)	12	(48)				14	(15)	9	(64)	5	(36)			
**T3+T4**	113	(79)	62	(55)	51	(45)	0.38	1.38	(0.68–2.82)	78	(85)	51	(65)	27	(35)	0.77	1.20	(0.36–4.04)
**Unclear**	5	(3)	2	(40)	3	(60)				ND^▲^	ND	ND	ND	ND	ND			
**Tumor stage (N) classification**																		
**N0**	91	(64)	48	(53)	43	(47)				44	(48)	28	(64)	16	(36)			
**N1**	47	(33)	27	(57)	20	(43)	0.60	1.21	(0.60–2.46)	48	(52)	32	(67)	16	(33)	0.76	1.143	(0.48–2.70)
**Unclear**	5	(3)	1	(20)	4	(80)				ND	ND	ND	ND	ND	ND			
**Tumor stage (TNM) classification**																		
**I+II**	91	(64)	47	(52)	44	(48)				47	(51)	30	(64)	17	(36)			
**III+IV**	47	(33)	28	(60)	19	(40)	0.38	1.38	(0.68–2.82)	45	(49)	30	(67)	15	(33)	0.78	1.13	(0.48–2.68)
**Unclear**	5	(3)	1	(20)	4	(80)				ND	ND	ND	ND	ND	ND			
**RASSF1A protein**																		
**positive**	17	(63)	6	(35)	11	(65)				14	(58)	5	(36)	9	(14)			
**negative**	10	(37)	7	(70)	3	(30)	0.09	0.23	(0.04–1.25)	10	(42)	10	(100)	0	(0)	**0.001**		

^**Φ**^: Histopathologically, the gross pathologic types were different for ESCC and GCA, in ESCC, 1: medullary; 2: fungating; 3: ulcerating; 4: constriction & intraluminal; in GCA, 1: protruding; 2: ulcerating; 3: infiltrating.

^▲^: Not detected.

### Correlations of the RASSF1A protein immunoreactivity and hypermethylation of *RASSF1A* gene

In ESCC, all the 7 cases with *RASSF1A* promoter methylation-positive tissues (7/10, 70%), showed completely lack of immunoreactivity for RASSF1A, similar results were observed for the matched adjacent normal tissue. Interestingly, of the 17 cases with *RASSF1A* promoter methylation-negative tissues, 11 cases showed positive immunoreactivity for RASSF1A (11/17, 65%). RASSF1A positive staining was negatively associated with *RASSF1A* promoter methylation in the ESCC (*P*=0.09, Fisher’s exact test) (Table 3).

In GCA, all the 10 cases with *RASSF1A* promoter methylation-positive tissues, RASSF1A protein expression was not detected (Table 3). But, of the 14 cases with *RASSF1A* promoter methylation-negative tissues, 9 cases showed RASSF1A positive expression (64%). There was a significant correlation between *RASSF1A* promoter hypermethylation and loss of RASSF1A protein expression in GCA (*P*=0.001, Fisher’s exact test).

### RASSF1A protein expression in ESCC and GCA patients

The positive expression of the RASSF1A protein was located in cell nucleus and cytoplasm. The positive immunostaining rate for RASSF1A in ESCC tissue was slightly lower than in adjacent normal tissues (17/27, 63% vs. 21/27, 78%). But, the difference was not significant (*P*=0.24) (Table 4). No association was found for RASSF1A protein expression and gender, age, cancer cell differentiation, infiltration degree and lymph node metastasis or clinical stages in ESCC (Table 5).

**Table 4:** RASSF1A protein expression in ESCC and GCA tissues and in normal tissues adjacent to the corresponding ESCC and GCA

**Tissues**	**N**	**Postive**		**Negative**		***P***	***OR***	***(95%CI)***
		**n**	**(%)**	**n**	**(%)**			
**ESCC**	27	17	(63)	10	(37 )	0.24	0.47	(0.13–1.67)
**ENOR**	23	18	(78)	5	(22)			
**GCA**	24	14	(58)	10	(42)	**0.03**	0.16	(0.03–0.83)
**GNOR**	20	18	(90)	2	(10)			

^#^: ESCC: esophageal squamous cell carcinoma, ENOR: esophageal normal epithelial tissue, GCA: gastric cardia adenocarcinoma, GNOR: gastric cardia normal epithelial tissue.

**Table 5:** Correlation between RASSF1A protein expression and clinicopathological parameters of ESCC and GCA patients

**Classification**		**ESCC (n=27)**								**GCA (n=24)**							
		**N**		**Positive**		**Negative**		***P***	**OR(95%CI)**	**N**		**Positive**		**Negative**		***P***	***OR(95%CI)***
		**n**	**(%)**	**n**	**(%)**	**n**	**(%)**			**n**	**(%)**	**n**	**(%)**	**n**	**(%)**		
**Gender**																	
	**Male**	18	(67)	10	(56)	8	(44)			14	(58)	6	(43)	8	(57)		
	**Female**	9	(33)	7	(78)	2	(22)	0.27	0.36(0.06–2.22)	10	(42)	8	(80)	2	(20)	0.08	0.19(0.03–1.23)
**Age(y)**																	
	**≤40**	12	(44)	10	(83)	2	(17)			10	(42)	9	(90)	1	(10)		
	**≥70**	15	(56)	7	(47)	8	(53)	0.06	5.71(0.92–35.48)	14	(58)	5	(36)	9	(64)	**0.02**	16.20(1.57–167.74)
**Differentiation classification**																	
	**High+Middle**	23	(85)	13	(57)	10	(43)			14	(58)	6	(43)	8	(57)		
	**Low**	4	(15)	4	(100)	0	(0)	0.10		10	(42)	8	(80)	2	(20)	0.08	0.19(0.03–1.23)
**Tumor stage (T) classification**																	
	**T1+T2**	6	(22)	4	(67)	2	(33)			2	(8)	1	(50)	1	(50)		
	**T3+T4**	21	(78)	13	(62)	8	(38)	0.57	1.88(0.22–15.93)	22	(92)	13	(59)	9	(41)	0.73	0.75(0.15–3.83)
**Tumor stage (N) classification**																	
	**N0**	20	(74)	13	(65)	7	(35)			15	(63)	9	(60)	6	(40)		
	**N1**	7	(26)	4	(57)	3	(43)	0.41	2.18(0.35–13.76)	9	(38)	5	(56)	4	(44)	0.83	0.83(0.16–4.44)
**Tumor stage (TNM) classification**																	
	**I+II**	20	(74)	12	(60)	8	(40)			21	(88)	12	(57)	9	(43)		
	**III+IV**	7	(26)	5	(71)	2	(29)	0.59	1.67(0.26–10.79)	3	(13)	2	(67)	1	(33)	0.76	1.50(0.12–19.24)

However, RASSF1A protein positive immunostaining rate in GCA tissues was much lower than in adjacent normal gastric cardia tissue (14/24, 58% vs. 22/24, 92%, *P=*0.02) (Table 4). Furthermore, RASSF1A expression in GCA patients over 70 years old was significantly higher than in those under 40 years old (58% vs. 42%; OR=16.20, 95% CI=1.57–167.74) (Table 5). No association was found for RASSF1A protein expression and gender, cancer cell differentiation, infiltration degree and lymph node metastasis in GCA (Table 5).

## Discussion

In the present study, we firstly investigated the *RASSF1A* A133S polymorphism and the risk to ESCC and GCA development on the patients from Linzhou, the high incidence area for both ESCC and GCA. Our results demonstrated that the existence of polymorphic allele of *RASSF1A* might participate in gastric cardia carcinogenesis. The carriers with Ser/Ser genotype (the homozygote for codon *RASSF1A* A133S) and with mutated T allele genotype (Ala/Ser+Ser/Ser genotype) could have 9-fold (OR=9.01, 95% CI=0.99–83.31) and 2-fold (OR=2.06, 95% CI=1.09–3.97) higher risk to GCA, respectively. In contrast, the presence of SNP A133S in *RASSF1A* seems to be not involved in esophageal squamous cell carcinogenesis (*P*=0.69). Histopathologically, the predominant type for gastric cardia is adenocarcinoma, but the predominant type for esophageal cancer is squmous cell carcinoma in Chinese population, which may partially explain the genetic risk difference observed in the present study. It has been reported that single nucleotide polymorphism at codon 133 of the *RASSF1* gene is preferentially associated with human lung adenocarcinoma risk, but not for human lung squamous cell carcinoma [11].

In this study, we also investigated *RASSF1A* promoter methylation in ESCC and GCA. Aberrant *RASSF1A* promoter methylation was found in 53% (76/143) of Chinese ESCC, significantly increased 5.9-fold higher the risk to ESCC (OR=5.90, 95% CI=2.78–12.52). It was apparently higher than in Korean ESCC (14%, 7/50) [18] and Japanese ESCC (24%, 13/55) [19]. Interestingly, another two reports also show higher frequency of *RASSF1A* promoter methylation in Chinese ESCC from Hong Kong (34%, 22/64) [20] and Hangzhou (48.5%, 32/66)[10], the high incidence area for ESCC in China. It is noteworthy that Chinese ESCC from low incidence area seems to have low frequency of *RASSF1A* promoter methylation (14.9% 22/64) [9]. Obviously, the *RASSF1A* promoter methylation may be interfered by genetic and environmental factors from different population. In this study, we found that the prevalence of *RASSF1A* promoter methylation and the risk to ESCC in ESCC patients with the age of more than 50 years old were higher than those with less than 50 years old (OR=3.11, 95% CI=1.10–8.73), which further indicate the impact of environmental factor in terms of exposure time on *RASSF1A* promoter methylation.

Of particular interest, we also demonstrated the similar hypermethylation of *RASSF1A* in GCA (65%, 60/92), the promoter hypermethylation of *RASSF1A* significantly increased 7.5-fold higher the risk to GCA (OR=7.5, 95% CI=2.78–20.23), which is very similar as in ESCC. It is noteworthy that these GCA patients enrolled in our study were from the same high incidence area as the ESCC patients, suggesting that there may be similar environmental carcinogenic factors involved in ESCC and GCA. GCA seems to occur together with ESCC in China and bears many similarities in terms of concurrent geographic distribution and environmental risk factors including nutritional deficiencies, low intake of vegetables and fruit, and low socioeconomic status [14]. Recent GWAS studies have demonstrated the similar genetic changes for ESCC and GCA in Chinese population [6]. Furthermore, our results is close to the findings of *RASSF1A* gene methylation in GCA patients from Hebei province (58.7%, 54/92) [21], another high incidence area for ESCC and GCA in TaiHang Mountain.

It is noteworthy that, in the present study, we also observed the *RASSF1A* promoter methylation in the normal esophageal and gastric cardia epithelial tissue adjacent to corresponding ESCC and GCA (in ESCC: 13%, 23/143; in GCA: 20%, 19/92). These histological normal tissue has been exposed to same carcinogenic factors with tumor tissues. The present results indicate that *RASSF1A* promoter methylation may be a promising early indicator for esophageal and gastric cardia carcinogenesis. Further studies are required to characterize the *RASSF1A* promoter methylation in esophageal and gastric cardia precancerous lesion.

Another interesting finding in this study is that *RASSF1A* promoter hypermethylation could significantly downregulated RASSF1A protein expression both in ESCC and GCA, further indicating the possible crucial role of *RASSF1A* promoter hypermethylation and protein expression in esophageal and gastric cardia carcinogenesis.

Finally, considering the obvious impact of environmental factors on DNA methylation, the advantage of our study is the large sample size with similar lifestyle background. All the ESCC and GCA patients and control subjects enrolled in this study were from the same high incidence area for both ESCC and GCA.

## Conclusions

In conclusion, this is the first study to reveal the interactions of *RASSF1A* polymorphism, promoter methylation and protein expression on the risk of esophageal and gastric cardia carcinogenesis. *RASSF1A* promoter hypermethylation could significantly increase 6-fold and 8-fold higher risk to ESCC and GCA development, respectively, and induce the inactivation of RASSF1A protein expression both in ESCC and GCA, which indicate that *RASSF1A* promoter hypermethylation may play an important role both in esophageal and gastric cardia carcinogenesis. The T allele (Ala/Ser + Ser/Ser genotype) of *RASSF1A* A133S could increase the risk to GCA development, but not to ESCC. Certainly, further work should be done to illustrate the mechanism of RASSF1A in the development of ESCC and GCA, and ultimately develop *RASSF1A* gene as one of the molecular biomarkers for high-risk subject screening and early detection for ESCC and GCA in future.

## Abbreviations

RASSF1A: Ras association domain family 1A gene; ESCC: Esophageal squamous cell carcinoma; SNP: Single nucleotide polymorphism; GCA: Gastric cardia adenocarcinoma; PCR: Polymerase chain reaction.

## Competing interests

All authors declare no competing financial interests.

## Authors’ contributions

LD Wang and SL Zhou conceived of the study, participated in the design of the study and drafted the manuscript. J Cui performed the statistical analysis and clinical data collection. ZM Fan and BC Liu carried out polymorphism examination. XM Li and J L Li participated in pathological finding analysis. SL Zhou and DY Zhang carried out the methylation detection. HY Liu, XK Zhao and X Song participated in the immunohistochemical analysis. R Wang, ZC Yan and HX Yi helped to worked out the methylation detection. All authors read and approved the final manuscript.

## Pre-publication history

The pre-publication history for this paper can be accessed here:

http://www.biomedcentral.com/1471-2407/13/259/prepub
